# Improving Sugarcane Biomass and Phosphorus Fertilization Through Phosphate-Solubilizing Bacteria: A Photosynthesis-Based Approach

**DOI:** 10.3390/plants14172732

**Published:** 2025-09-02

**Authors:** Hariane Luiz Santos, Gustavo Ferreira da Silva, Melina Rodrigues Alves Carnietto, Gustavo Ferreira da Silva, Caio Nascimento Fernandes, Lusiane de Sousa Ferreira, Marcelo de Almeida Silva

**Affiliations:** 1 Laboratory of Ecophysiology Applied to Agriculture (LECA), Department of Crop Production, School of Agricultural Sciences, Sâo Paulo State University (UNESP), Botucatu 18610-034, SP, Brazil; gustavo.ferreira-silva@unesp.br (G.F.d.S.); melina.carnietto@unesp.br (M.R.A.C.); lusiane.ferreira@unesp.br (L.d.S.F.); 2 Agricultural Sciences Center, Department of Biotechnology and Plant and Animal Production, Federal University of São Carlos (UFSCar), Araras 13600-970, SP, Brazil; gustavo.ferreira@ufscar.br; 3 Department of Rural Engineering and Socioeconomics, School of Agricultural Sciences, Sâo Paulo State University (UNESP), Botucatu 18610-034, SP, Brazil; cn.fernandes@unesp.br

**Keywords:** gas exchange, leaf acid phosphatase, phosphate-solubilizing bacteria, photochemical efficiency, *Saccharum* spp., shoot dry mass

## Abstract

Phosphorus (P) is essential for sugarcane growth but often presents low agricultural use efficiency. This research evaluated the effects of *Bacillus velezensis* UFV 3918 (*Bv*), applied alone or with monoammonium phosphate (MAP), on sugarcane’s physiological, biochemical, and biomass variables. Six treatments were tested in a completely randomized design: absolute control (AC), commercial control (CC, full MAP dose), *Bv* alone, and *Bv* combined with 1/3, 2/3, or full MAP dose. *B. velezensis (Bv)* and *Bv* + 1/3 MAP increased soil P availability by 22%, correlating strongly with physiological, biochemical, and shoot biomass variables. These treatments boosted total chlorophyll content (11.4%), electron transport rate (28.5%), and photochemical quenching (16.9%), resulting in higher photosynthetic efficiency. Compared with CC, net CO_2_ assimilation, stomatal conductance, and carboxylation efficiency increased by 49.0%, 35.4%, and 72.9%, respectively. Additionally, amino acid content and leaf acid phosphatase activity rose by 12.1% and 13.8%. Key traits associated with biomass production included stomatal density (abaxial face), chlorophyll content, electron transport rate, intercellular CO_2_ concentration, and leaf acid phosphatase activity. The results highlight the potential of *Bv* UFV 3918, particularly with reduced MAP doses, to improve sugarcane photosynthesis and biomass accumulation, offering a sustainable and cost-effective fertilization strategy.

## 1. Introduction

Sugarcane is a vital global crop and the primary source of sugar and ethanol [1]. Brazil is the world’s leading producer, harvesting 713.2 million tons over 8.33 million hectares in the 2023/2024 season [2]. Achieving high yields depends heavily on adequate fertilization, with phosphorus (P) management being particularly critical due to its role as a limiting nutrient in many agricultural systems [3,4]. Phosphorus is essential for plant growth and reproduction, contributing to cell division, photosynthesis, and respiration, which are fundamental for early root establishment, sprouting, and tillering. Insufficient P supply can negatively impact photosynthetic activity, sucrose synthesis, stalk productivity, and sugarcane field longevity [5,6,7,8,9,10]. Although many soils contain substantial P reserves (100–3000 mg P kg^–1^), only a small fraction (0.1–10 μM) is readily available to plants [11,12]. Approximately 4% of total soil P is accessible in orthophosphate [13], while inorganic P constitutes around 35–70% of total soil phosphorus [3], highlighting the importance of strategies that enhance P bioavailability for sugarcane cultivation.

Phosphorus has the lowest use efficiency among macronutrients in agricultural production due to its strong interactions with soil components [14,15,16]. This limitation is particularly pronounced in tropical regions, where most soluble phosphate fertilizers applied to the soil dissolve but are rapidly retained in the solid phase through adsorption by iron and aluminum minerals, reducing P availability for plant growth [17,18,19]. In sugarcane cultivation, where soils often exhibit high P-fixing capacity, substantial fertilizer inputs are required to meet crop demands [20]. Given the rising global demand for sugar and bioethanol, improving the efficiency of phosphate fertilization and enhancing both the photochemical and biochemical components of photosynthesis are critical to producing sugarcane more economically and sustainably.

Phosphate-solubilizing bacteria (PSB) are a sustainable approach to increasing P availability in agricultural systems [13,21,22,23,24] while also enhancing phosphorus use efficiency in the soil–plant system [25,26]. These microorganisms are key in converting inorganic and organic P forms into bioavailable compounds [27,28]. *Bacillus* species are particularly abundant in the rhizosphere and stand out for their potential as efficient P solubilizers [29,30].

Beyond P solubilization, *Bacillus* species can favor plant photosynthetic capacity by increasing chlorophyll content, contributing to higher photochemical efficiency [31,32]. This effect may occur directly by producing phytohormones, siderophores, or other metabolites that stimulate chlorophyll biosynthesis, and indirectly due to improved nutrient uptake supporting chlorophyll formation [33]. Moreover, PSB can enhance the synthesis of osmoregulatory substances [34], influencing stomatal conductance and cellular tolerance to dehydration [35,36,37], promoting greater water use efficiency [36,38] and higher carboxylation efficiency [38]. In this way, inoculation with bacteria can favor the growth and productivity of sugarcane [23,39,40].

This study aimed to assess sugarcane’s physiological, biochemical, and production responses up to 180 days after planting, using *Bacillus velezensis* strain UFV 3918, applied alone or in combination with mono ammonium phosphate (MAP). We hypothesized that inoculating sugarcane buds with *B. velezensis* UFV 3918 could reduce the need for MAP application while maintaining or enhancing photosynthetic efficiency and biomass productivity.

## 2. Results

### 2.1. Physiological Assessments

**Figure 1:**
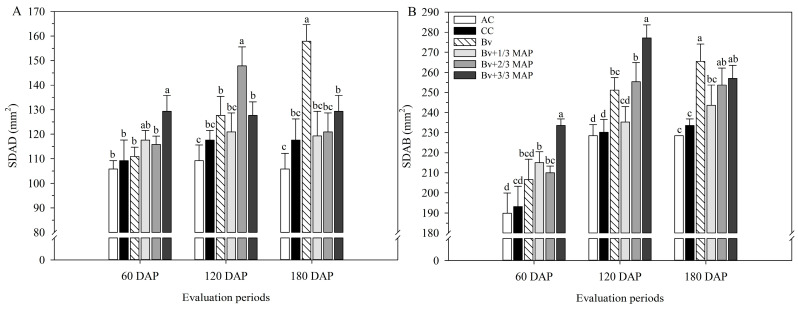
Adaxial stomatal density (SDAD) (**A**) and abaxial stomatal density (SDAB) (**B**) of sugarcane leaves under treatments with and without inoculation of *Bacillus velezensis* UFV 3918 (*Bv*) and doses of mono ammonium phosphate (MAP), at 60, 120, and 180 DAP. Averages followed by the same letter do not differ according to Tukey’s test at 5% probability. The error bars express the standard deviation of the mean (*n* = 4). AC: absolute control (without MAP); CC: commercial control (3/3 MAP—100% of recommended MAP dose, without *Bv*).

Considering adaxial stomatal density (SDAD), at 60 DAP, the highest value was found in *Bv* + 3/3 MAP, which was 18.4% higher than in CC (Figure 1A). At 120 DAP, CC, *Bv*, *Bv* + 1/3 MAP, and *Bv* + 3/3 MAP resulted in similar SDAD, while *Bv* + 2/3 MAP increased the SDAD by 25.7% compared with the CC. At 180 DAP, the highest SDAD was observed in *Bv*, representing 34.3% and 28.2% increases compared with CC and the other inoculated treatments, respectively (Figure 1A). Similar performance was observed in abaxial stomatal density (SDAB) at 60 DAP, where *Bv* + 3/3 MAP provided increases of 20.9% and 10.9% compared with CC and the other inoculated treatments, respectively (Figure 1B). At 120 and 180 DAP, all treatments with *B. velezensis* resulted in SDAB equal to or greater than CC. *Bv* and *Bv* + 2/3 MAP provided average increases of 10.0% and 11.2% in SDAB compared with CC at 120 and 180 DAP, respectively (Figure 1B).

**Figure 2:**
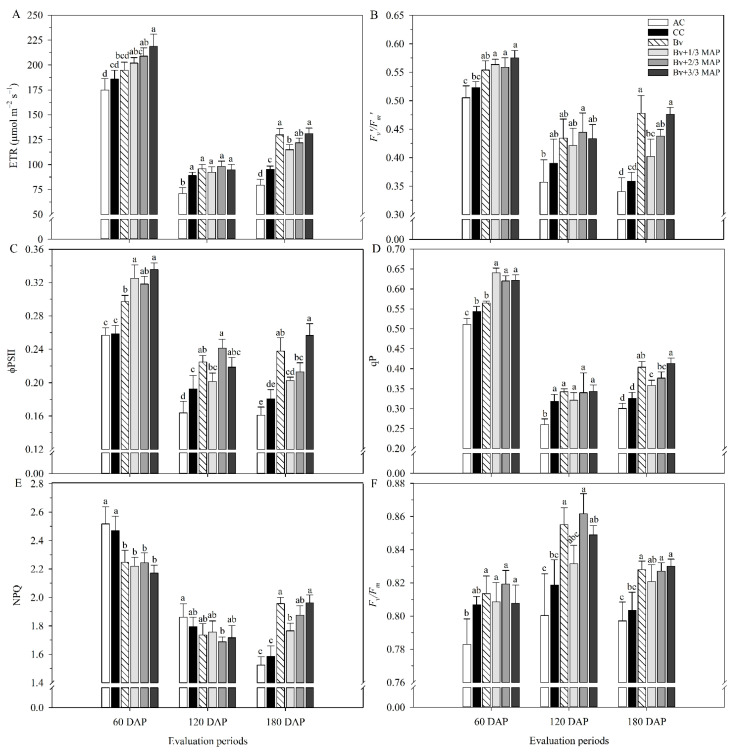
Relative electron transport rate (ETR) (**A**), potential quantum yield of PSII (*F_v_′*/*F_m_′*) (**B**), effective quantum yield of linear electron flow through PSII (φPSII) (**C**), photochemical quenching (qP) (**D**), non-photochemical quenching (NPQ) (**E**), maximum variable quantum yield of PSII (*F_v_*/*F_m_*) (**F**) of sugarcane plants under treatments with and without inoculation of *Bacillus velezensis* UFV 3918 (*Bv*) and doses of mono ammonium phosphate (MAP), at 60, 120, and 180 DAP. Averages followed by the same letter do not differ according to Tukey’s test at 5% probability. The error bars express the standard deviation of the mean (*n* = 4). AC: absolute control (without MAP); CC: commercial control (3/3 MAP—100% of recommended MAP dose, without *Bv*).

The lowest performance regarding the variables related to chlorophyll *a* fluorescence was observed in AC (Figure 2). There was a general downward trend in ETR, *F_v_′*/*F_m_′*, φPSII, qP, and NPQ between 60 and 180 DAP (Figure 2A–E). Furthermore, at 120 DAP, there was no difference between the inoculated treatments and CC for ETR, *F_v_′*/*F_m_′*, qP, and NPQ (Figure 2A–E).

At 60 DAP, the highest ETR was observed in *Bv* + 2/3 MAP and *Bv* + 3/3 MAP, which provided an average increase of 15% compared with CC. At 180 DAP, *Bv* and *Bv* + 1/3 MAP increased the ETR by 28.5% compared with CC (Figure 2A). Regarding *F_v_′*/*F_m_′*, at 60 DAP, the highest values were observed in *Bv* + 1/3 MAP, 2/3 MAP, and 3/3 MAP, which provided an average increase of 8.2% compared with CC (Figure 2B). At 180 DAP, there was a 33.1% increase in the *F_v_′*/*F_m_′* of *Bv* compared with CC.

For φPSII, *Bv* + 1/3, 2/3, and 3/3 MAP provided an average increase of 25.9% compared with CC at 60 DAP. In contrast, *Bv* increased φPSII by 17.2% and 31.5% compared with CC at 120 and 180 DAP, respectively, but was similar to *Bv* + 3/3 MAP in both periods (Figure 2C). At 60 DAP, the highest qP was found in *Bv* + 1/3, 2/3, and 3/3 MAP, which provided an average increase of 15.6% compared with CC (Figure 2D). At 180 DAP, there was a 23.9% increase in the qP of *Bv* compared with CC. Still, *Bv* was similar to *Bv* + 3/3 MAP, and in general, in the same period, treatments inoculated with *B. velezensis* provided an average increase of 19.0% in qP compared with CC.

On average, AC and CC provided NPQ 12.2% higher than those observed in plants inoculated with *B. velezensis* at 60 DAP. In contrast, at 180 DAP, inoculated plants provided an average increase of 21.6% in NPQ compared with non-inoculated plants, emphasizing *Bv*, *Bv* + 2/3 MAP, and *Bv* + 3/3 MAP (Figure 2E). At 120 DAP, *Bv* and *Bv* + 2/3 MAP provided an average increase of 4.8% in *F_v_*/*F_m_* compared with CC, and at 180 DAP, *Bv*, *Bv* + 2/3 MAP, and *Bv* + 3/3 MAP provided an average increase of 3.0% in this variable compared with CC (Figure 2F).

**Figure 3:**
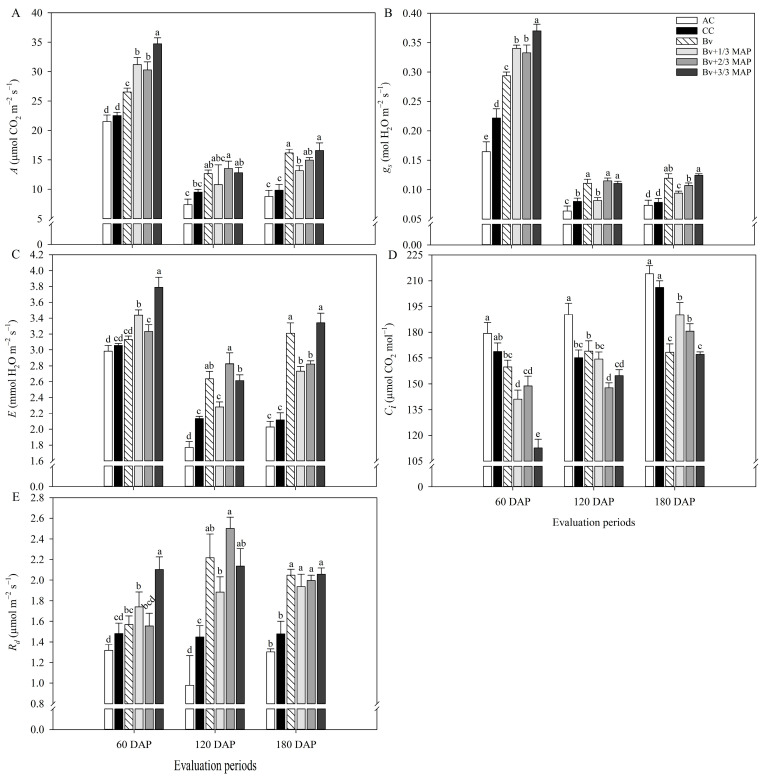
Net CO_2_ assimilation rate (*A*) (**A**), stomatal conductance (*g_s_*) (**B**), transpiration rate (*E*) (**C**), intercellular CO_2_ concentration (*C_i_*) (**D**), and night respiration (*R_d_*) (**E**) of sugarcane plants under treatments with and without inoculation of *Bacillus velezensis* UFV 3918 (*Bv*) and doses of mono ammonium phosphate (MAP), at 60, 120, and 180 DAP. Averages followed by the same letter do not differ according to Tukey’s test at 5% probability. The error bars express the standard deviation of the mean (*n* = 4). AC: absolute control (without MAP); CC: commercial control (3/3 MAP—100% of recommended MAP dose, without *Bv*).

**Figure 4:**
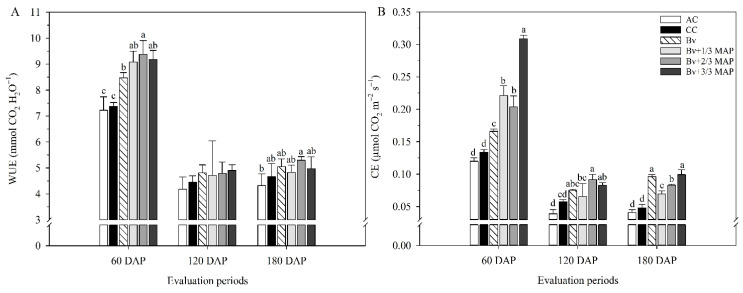
Instantaneous water use efficiency (WUE) (**A**) and instantaneous carboxylation efficiency (CE) (**B**) of sugarcane plants under treatments with and without inoculation of *Bacillus velezensis* UFV 3918 (*Bv*) and doses of mono ammonium phosphate (MAP), at 60, 120, and 180 DAP. Averages followed by the same letter do not differ according to Tukey’s test at 5% probability. The error bars express the standard deviation of the mean (*n* = 4). AC: absolute control (without MAP); CC: commercial control (3/3 MAP—100% of recommended MAP dose, without *Bv*).

As well as chlorophyll *a* fluorescence variables, there were decreases in *A*, *g_s_*, and *E* and an increase in *C_i_* between 60 and 180 DAP (Figure 3), contributing to the reduction in WUE and EC throughout this period (Figure 4). This may have resulted from the plants’ aging and the reduction in the average temperature observed throughout the evaluations (Figure 12).

AC also had the lowest physiological performance in gas exchange throughout the cycle (Figure 3). At 60 DAP, *Bv* + 3/3 MAP had the best gas exchange performance. However, throughout the evaluations, treatments with *B. velezensis* and reduced phosphate doses improved photosynthetic performance, even equaling *Bv* + 3/3 MAP, with *Bv* standing out (Figure 3).

At 60 DAP, *Bv* + 1/3 MAP increased *A* by 38.4% compared with CC, while *Bv* and *Bv* + 1/3 MAP provided average increases of 23.3% and 49.0% in *A* compared with CC, at 120 and 180 DAP, respectively (Figure 3A). Furthermore, *Bv* was similar to *Bv* + 2/3 MAP and *Bv* + 3/3 MAP in both periods. Regarding *g_s_*, *Bv* and *Bv* + 1/3 MAP promoted average increases of 42.8% and 35.4% compared with CC at 60 and 180 DAP, respectively. There was no difference between *Bv*, *Bv* + 2/3 MAP, and *Bv* + 3/3 MAP for *g_s_* at 120 DAP, but these treatments increased *g_s_*, on average, by 40% compared with CC (Figure 3B).

*Bv* had *E* similar to CC at 60 DAP but increased this variable by 23.6% and 51.6% at 120 and 180 DAP, respectively (Figure 3C), due to the higher *g_s_* and *A* observed during this period. Regarding *C_i_*, there was no difference between *Bv* and CC at 60 and 120 DAP; however, at 180 DAP, *Bv* resulted in *C_i_* 18.3% lower than CC and similar to *Bv* + 3/3 MAP (Figure 3D). *Bv* + 1/3 MAP provided *C_i_* similar to CC at 120 DAP, but decreases of 16.3% and 7.8% were observed in the *C_i_* of *Bv* + 1/3 MAP compared with CC at 60 and 180 DAP, respectively (Figure 3D).

At 60 DAP, *Bv* + 3/3 MAP provided the highest *R_d_*, with 42.1% and 29.8% increases compared with CC and the other treatments inoculated with *B. velezensis*, respectively (Figure 3E). Plants inoculated with the UFV 3918 strain showed average gains of 50.8% and 35.8% in *R_d_* compared with CC at 120 and 180 DAP, respectively.

At 60 DAP, *Bv* and *Bv* + 1/3 MAP had WUE, on average, 14.8% higher than CC but similar to *Bv* + 3/3 MAP. However, there was no difference between the inoculated treatments and CC at 120 and 180 DAP (Figure 4A). *Bv*, *Bv* + 1/3, 2/3, and 3/3 MAP increased CE by 23.9%, 64.9%, 52.2%, and 130.6%, respectively, compared with CC at 60 DAP. At 180 DAP, they provided increases of 100%, 43.8%, 72.9%, and 106.2% in CE, respectively, compared with CC (Figure 4B).

### 2.2. Biochemical Assessments

**Figure 5:**
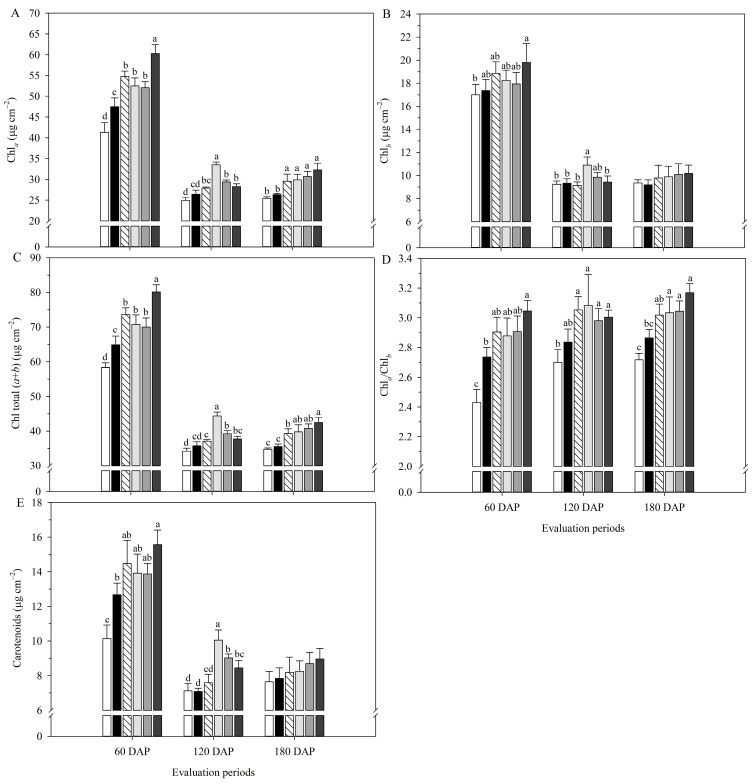
Contents of chlorophyll a (Chl*_a_*) (**A**), chlorophyll b (Chl*_b_*) (**B**), and total chlorophyll (Chl total) (**C**), Chl*_a_*/Chl*_b_* ratio (**D**), and carotenoid content (**E**) of sugarcane leaves under treatments with and without inoculation of *Bacillus velezensis* UFV 3918 (*Bv*) and doses of mono ammonium phosphate (MAP), at 60, 120, and 180 DAP. Averages followed by the same letter do not differ according to Tukey’s test at 5% probability. The error bars express the standard deviation of the mean (*n* = 4). AC: absolute control; CC: commercial control. AC: absolute control (without MAP); CC: commercial control (3/3 MAP—100% of recommended MAP dose, without *Bv*).

There were decreases in the contents of chlorophyll *a* (Chl*_a_*), chlorophyll *b* (Chl*_b_*), total chlorophyll (Chl total), and carotenoids between 60 DAP and 180 DAP because of the plants’ aging, and the lowest contents of photosynthetic pigments throughout the period were verified in the AC (Figure 5). At 60 DAP, *Bv* + 3/3 MAP performed the best in photosynthetic pigments. Still, throughout the evaluations, the combination of *Bv* and reduced MAP doses allowed it to equate to *Bv* + 3/3 MAP, with *Bv* and *Bv* + 1/3 MAP standing out.

*Bv* + 3/3 MAP increased Chl*_a_* content by 26.9% and 13.5% compared with CC and the other treatments inoculated with *B. velezensis*, respectively, at 60 DAP. At 120 DAP, *Bv* + 1/3 MAP increased this variable by 26.7% compared with CC. At 180 DAP, plants inoculated with *B. velezensis* had Chl*_a_* content, on average, 16.3% higher than CC (Figure 5A). Regarding Chl*_b_* content, there was no difference between CC and *Bv* + MAP doses at 60 DAP; however, at 120 DAP, *Bv* + 1/3 MAP increased Chl*_b_* content by 16.8% compared with CC (Figure 5B).

*Bv, Bv* + 1/3 MAP, and *Bv* + 2/3 MAP provided average increases of 10.1% and 12.5% compared to CC at 60 and 180 DAP, respectively. At 120 DAP, *Bv* + 1/3 MAP increased this variable by 24.2% compared with CC (Figure 5C). *Bv*, *Bv* + 1/3 MAP, and *Bv* + 2/3 MAP provided Chl*_a_*/Chl*_b_* similar to CC at 60 and 120 DAP, while *Bv* + 1/3 MAP increased this variable by 5.6% compared with CC at 180 DAP (Figure 5D).

Considering the carotenoid content, there was no difference between treatments inoculated with *B. velezensis* at 60 DAP, but *Bv* + 3/3 MAP provided a 22.9% increase in this variable compared with CC (Figure 5E). At 120 DAP, *Bv* + 1/3 MAP and *Bv* + 2/3 MAP increased the carotenoid content by 41.7% and 27.2% compared with CC, respectively. In comparison, there was no difference between treatments at 180 DAP.

**Figure 6:**
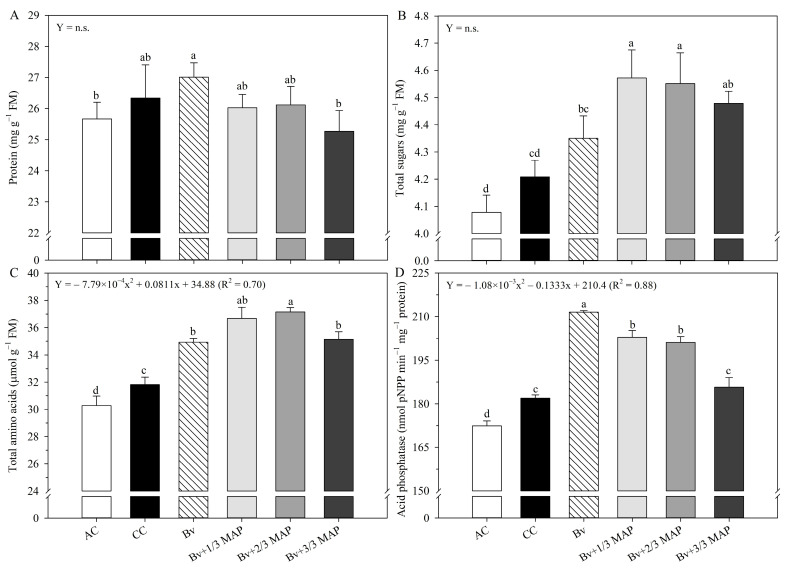
Protein content (**A**), total soluble sugars (**B**), total amino acids (**C**), and acid phosphatase activity (**D**) in sugarcane leaves under treatments with and without inoculation of *Bacillus velezensis* UFV 3918 (*Bv*) and doses of mono ammonium phosphate (MAP). Averages followed by the same letter do not differ according to Tukey’s test at 5% probability. The error bars express the standard deviation of the mean (*n* = 4). The regression equations and R^2^ refer to the association between *Bv* and MAP doses at a 5% significance level. AC: absolute control (without MAP); CC: commercial control (3/3 MAP—100% of recommended MAP dose, without *Bv*).

There was no difference in protein content between CC and *Bv* + MAP doses (Figure 6A), and no regression model was fitted for this variable. The highest total sugar (TS) contents were observed in *Bv* + 1/3 MAP and *Bv* + 2/3 MAP, which provided average increases of 4.8%, 9.3%, and 11.5% compared to *Bv*, CC, and AC, respectively (Figure 6B). There was no regression adjustment of *Bv* + MAP doses for TS content.

The highest total amino acid (TAC) contents were observed under *Bv* + MAP doses. *Bv*, *Bv* + 1/3 MAP, 2/3 MAP, and 3/3 MAP provided increases of 9.8%, 14.5%, 16.4%, and 10.3% in TAC content, respectively, compared with CC (Figure 6C). *Bv* + MAP doses raised TAC content up to 66% of the MAP dose, followed by a tendency to decrease with 100% MAP (0.70 *).

Regarding leaf acid phosphatase (LAP) activity, *Bv* and *Bv* + reduced MAP doses (*Bv* + 1/3 MAP and *Bv* + 2/3 MAP) provided the highest enzymatic activities, with average increases of 16.4% and 10.6%, respectively, compared with CC (Figure 6D). As *Bv* and increasing doses of MAP were associated, there was a reduction in LAP activity (0.88 *).

### 2.3. Phosphorus Content in the Soil and Shoot

The inoculated treatments had the highest soil P contents, with *Bv*, *Bv* + 1/3 MAP, and *Bv* + 3/3MAP providing increases of 26.2%, 17.8%, and 23.1%, respectively, compared with CC (Table 1). It is worth highlighting that *Bv* increased the P content in the soil by 13.6% compared with *Bv* + 2/3 MAP, similar to *Bv* + 3/3MAP. Considering *Bv+MAP* doses, there was a reduction in P content from 0 to 66% of the MAP dose, followed by an increase at the full MAP dose (0.70 *) (Table 1).

Considering the shoot P accumulation (PAc), although there was no statistical difference between *Bv* + 1/3, 2/3, and 3/3 MAP and CC, *Bv* provided increases of 29%, 11%, and 11% in PAc compared with AC, CC, and *Bv* + 1/3 MAP, respectively (Table 1). Regarding the *Bv+MAP* doses, there was a decrease in PAc from 0 to 33% of the MAP dose, followed by a slight increase at 66% and 100% of the MAP dose (0.70 *).

### 2.4. Shoot Biomass

**Figure 7:**
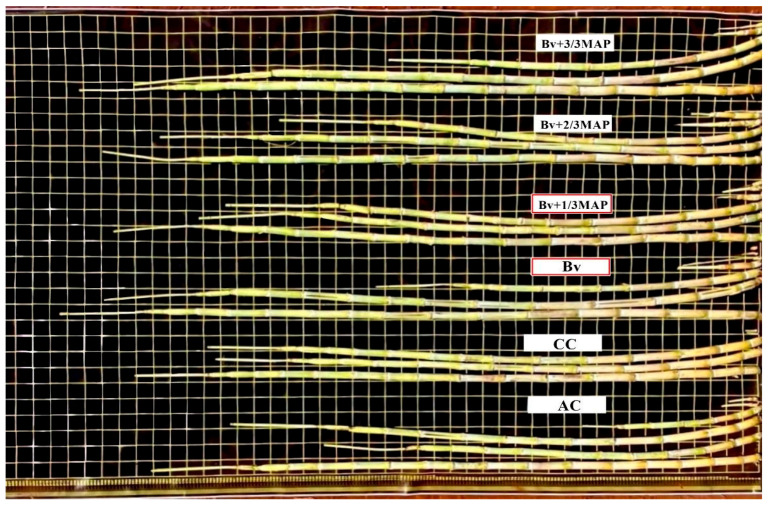
Visual appearance of sugarcane plant stalks under treatments with and without inoculation with *Bacillus velezensis* UFV 3918 (*Bv*) and mono ammonium phosphate (MAP) doses. AC: absolute control (without MAP); CC: commercial control (3/3 MAP—100% of recommended MAP dose, without *Bv*).

**Figure 8:**
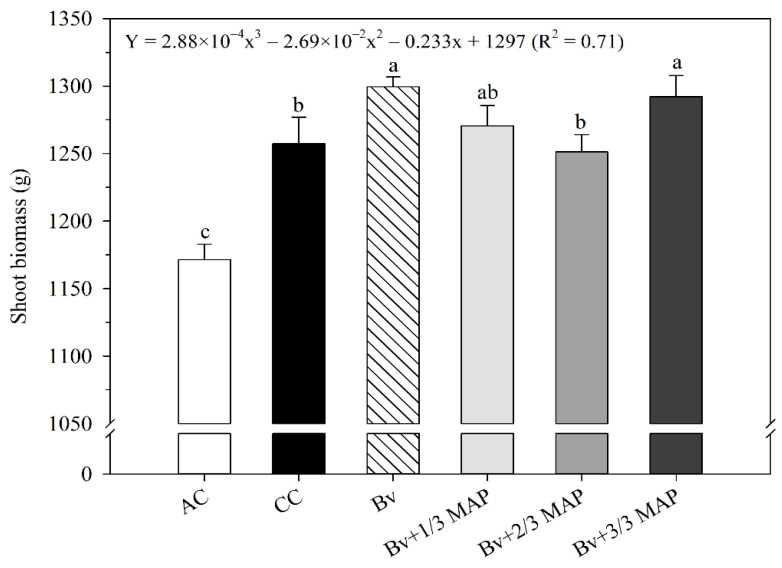
Shoot biomass (SB) of sugarcane plants at 180 DAP, under treatments with and without inoculation of *Bacillus velezensis* UFV 3918 (Bv) and doses of mono ammonium phosphate (MAP). Averages followed by the same letter do not differ according to Tukey’s test at 5% probability. The error bars express the standard deviation of the mean (*n* = 4). The regression equations and R^2^ refer to the association between *Bv* and MAP doses at a 5% significance level. AC: absolute control (without MAP); CC: commercial control (3/3 MAP—100% of recommended MAP dose, without *Bv*).

Using *B. velezensis* increased stalk growth (Figure 7), especially without MAP association, equivalent to the association of *Bv* + 3/3 MAP. Sugarcane shoot biomass was measured to accurately demonstrate the impact of *B. velezensis* on production (Figure 8).

Sugarcane plants showed different responses to biomass production between the treatments inoculated and non-inoculated with *B. velezensis* and different MAP doses (Figure 8). The highest shoot biomass (SB) was observed in *Bv* and *Bv* + 3/3 MAP. However, *Bv* + 1/3 MAP and *Bv* + 2/3MAP were equivalent to CC, showing that inoculation with the UFV 3918 strain allowed reducing the P dose without harming biomass production (Figure 8). *Bv* provided 3.2% and 4% higher SB than CC and *Bv* + 2/3 MAP, respectively. Regarding *Bv* + MAP doses, as the P dose increased, there was a subtle decrease in SB up to 66% of the recommended MAP dose, followed by a slight increase with 100% of the MAP dose (0.71 *).

### 2.5. Principal Component Analysis

Eigenvalues and their corresponding eigenvectors were derived from the correlation matrix of variable pairs within each group (stomatal density, photochemistry, gas exchange, photosynthetic pigments, and leaf biochemistry) for principal component analysis.

**Figure 9:**
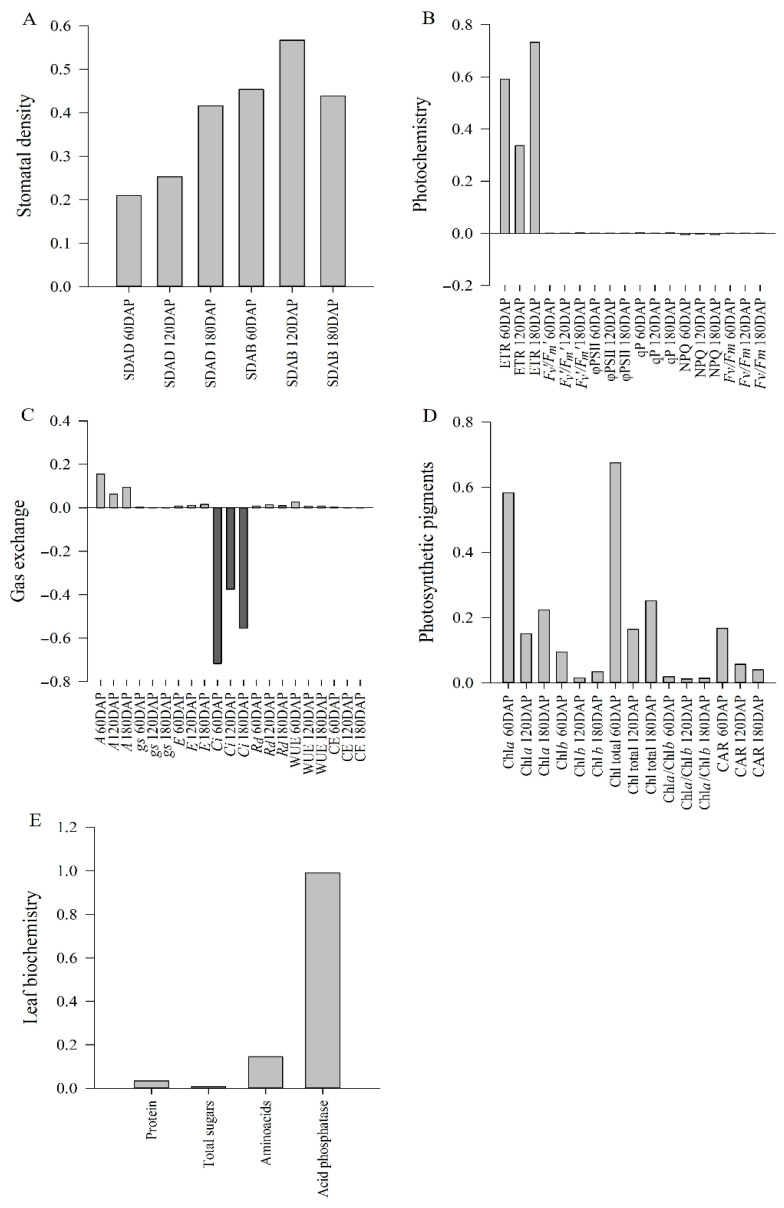
Variable loads of the components: stomatal density (**A**), photochemistry (**B**), gas exchange (**C**), photosynthetic pigments (**D**), and leaf biochemistry (**E**). Light gray indicates a positively charged variable; dark gray indicates a negatively charged variable. SDAD: adaxial stomatal density; SDAB: abaxial stomatal density; ETR: relative electron transport rate; *F_v_′*/*F_m_′*: potential quantum yield of PSII; φPSII: effective quantum yield of linear electron flow through PSII; qP: photochemical quenching, NPQ: non-photochemical quenching; *F_v_*/*F_m_*: maximum variable quantum yield of PSII; *A*: net CO_2_ assimilation rate; *g_s_*: stomatal conductance; *E*: transpiration rate; *C_i_*: intercellular CO_2_ concentration; *R_d_*: night respiration; WUE: instantaneous water use efficiency; CE: instantaneous carboxylation efficiency; chlorophyll *a* content: Chl*_a_*; chlorophyll *b* content: Chl*_b_*; carotenoid content: CAR.

The first principal component accounted for over 70% of the variance across all variable groups (Table 2) and was solely used to interpret the results. Among all the variables analyzed, stomata density on the abaxial surface at 120 DAP (SDAB 120 DAP), electron transport rate at 180 DAP (ETR 180 DAP), intercellular CO_2_ concentration (*C_i_*) at 60 and 180 DAP, chlorophyll *a* and total chlorophyll content at 60 DAP (Chl*_a_* 60 DAP and Chl total 60 DAP), and leaf acid phosphatase activity were the characteristics that explained the most of the respective components (Figure 9), with loadings of 0.56, 0.73, –0.71, –0.55, 0.58, 0.67, and 0.98, respectively.

**Figure 10:**
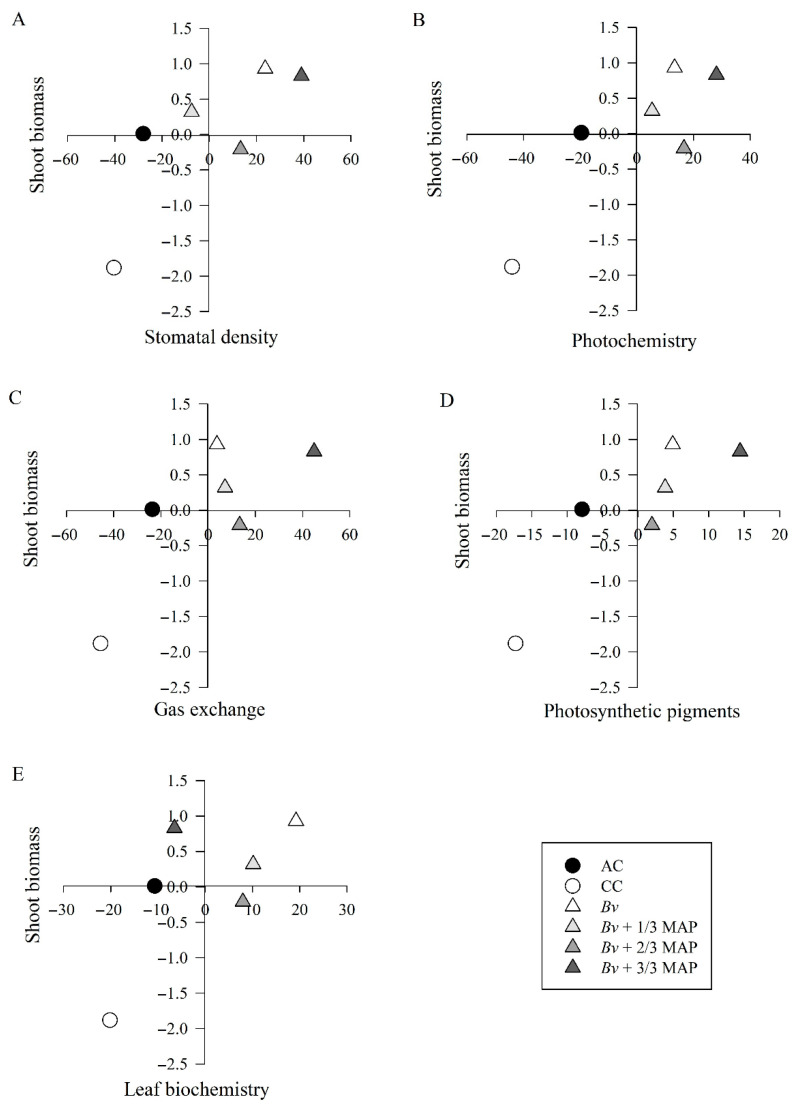
Dispersion of observations in the first component of stomatal density (**A**), photochemistry (**B**), gas exchange (**C**), photosynthetic pigments (**D**), and leaf biochemistry (**E**). AC: absolute control (without mono ammonium phosphate—MAP); CC: commercial control (3/3 MAP—100% of recommended MAP dose, without *Bv*); *Bacillus velezensis* UFV 3918 (*Bv*); *Bv* + 1/3 MAP; *Bv* + 2/3 MAP; *Bv* + 3/3 MAP.

The two-dimensional dispersion of the treatments showed differences between the inoculated (*Bv*, *Bv* + 1/3 MAP, *Bv* + 2/3 MAP, and *Bv* + 3/3 MAP) and non-inoculated (AC and CC) treatments for all variable groups. Plants inoculated only with *B. velezensis* (*Bv*) showed a higher potential for shoot biomass production than those without inoculation and similar to those inoculated in association with the highest MAP dose (*Bv* + 3/3 MAP) (Figure 10). Shoot biomass production in *Bv* (1297.02 g plant^−1^) was associated with higher SDAB (Figure 9A), higher ETR (Figure 9B), lower *C_i_* and higher *A* (Figure 9C), higher Chl*_a_* and total Chl contents (Figure 9D), and higher leaf acid phosphatase activity (Figure 9E).

**Figure 11:**
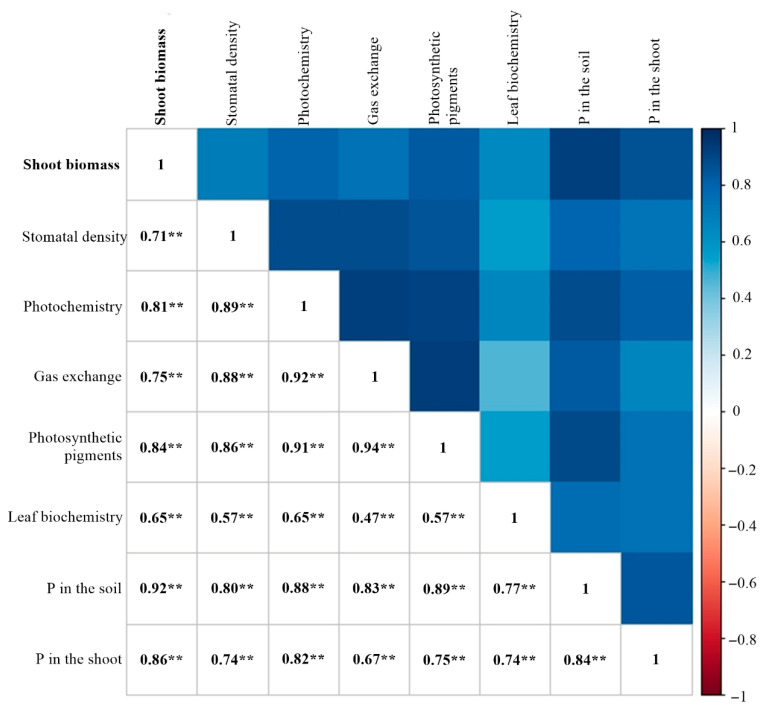
Pearson’s correlation between the first principal components of each variable group. ** indicates a significant correlation at *p* ≤ 0.01.

Based on Pearson’s correlation analysis (Figure 11), it was observed that the variable groups stomatal density (0.71), photochemistry (0.81), gas exchange (0.75), photosynthetic pigments (0.84), and leaf biochemistry (0.65) showed a significant correlation (*p* ≤ 0.01) with shoot biomass production (Figure 11). In addition, all sets of variables had a significant positive correlation.

In general, higher stomatal density was associated with higher electron transport rates, lower intercellular CO_2_ concentrations and higher net CO_2_ assimilation rates, higher photosynthetic pigment content, and higher leaf acid phosphatase activity, all of which contributed to higher biomass production (Figure 9 and Figure 11).

## 3. Discussion

*Bacillus* spp. strains are widely recognized as efficient phosphate-solubilizing bacteria (PSBs) [41,42,43,44]. Beyond enhancing plant growth, yield, and soil fertility [43,45,46], these microorganisms possess notable advantages, including inherent stability, resilience to adverse environmental conditions, and long shelf life [47,48,49], making them suitable for agricultural applications. Several studies have shown that combining PSBs with P fertilizers can reduce soil P adsorption and enhance P availability [45,50,51,52,53]. However, the dynamics between PSBs and reduced P doses remain an open research area.

Our findings shed light on sugarcane development during the first six months of growth, revealing that *B. velezensis* UFV 3918, whether applied alone or combined with reduced MAP doses, improved physiological, biochemical, and production traits.

Soil P status significantly influences plant metabolism, root exudation, and soil carbon availability for microorganisms [54,55]. Consequently, P fertilization can substantially alter P renewal efficiency [56] and regulate microbial communities as well as bacterial genes encoding enzymes involved in the P renewal cycle [57]. In unfertilized soils, PSBs typically increase P solubilization activity due to restricted inorganic P availability [58,59]. However, P fertilization may change the abundance of specific bacterial families [60,61]. Thus, the high initial soil P content (61.8 mg dm^−3^) may have limited the apparent synergistic effect between MAP and PSB. Readily available P often suppresses microbial phosphate-solubilization pathways and reduces the selective advantage of inoculated *Bacillus* strains [60,61].

This helps explain why *B. velezensis* without MAP performed better than its combination with 1/3 or 2/3 MAP for most variables. The superior results obtained with the full MAP dose (*Bv* + 3/3 MAP) probably reflect the high solubility of MAP itself rather than a synergistic effect with the bacterium. Thus, our data suggest that 2/3 MAP may represent a threshold beyond which chemical fertilization masked bacterial benefits. As *B. velezensis* + MAP doses were combined, there was an increase in the plant photosynthetic activity compared to the commercial control, but these treatments did not outperform *Bv*. Accordingly, the greater P availability observed under *B. velezensis* without MAP reflects stimulation of solubilization pathways under high baseline P conditions. This finding reinforces the potential of PSBs to reduce P inputs while maintaining plant performance.

Inoculation also influenced leaf physiology. PSBs are frequently associated with increased leaf area [62,63], which favors light interception and photosynthesis. Photosynthetic pigment content in leaves indicates photosynthetic capacity and physiological plant status [64,65,66,67]. Chlorophylls are the primary pigments that capture light energy and drive electron transport, thereby sustaining photosynthetic reactions [68,69]. Carotenoids complement this process by broadening the light absorption spectrum, protecting against photo-oxidation through the xanthophyll cycle, and stabilizing photosynthesis [68,69,70,71].

Enebe and Babalola [72] note that plant growth-promoting bacteria (PGPB) support the stability of photosynthetic pigments. In this study, inoculated plants exhibited higher contents of Chl*_a_*, total Chl, and an increased Chl*_a_*/Chl*_b_* ratio, suggesting enhanced chlorophyll biosynthesis. Elevated Chl*_a_* levels, the primary pigment responsible for converting light energy into chemical energy, relative to Chl*_b_*, which primarily absorbs and stores light energy, indicate improved light energy utilization in inoculated plants. Notably, Chl*_a_* emerged as one of the most influential variables contributing to shoot biomass production. Similar increases in photosynthetic pigment content following inoculation with *Bacillus* strains have been reported in crops such as sugarcane [38,73], corn [74,75,76], wheat [77], and soybean [78].

In contrast, non-inoculated plants without phosphate fertilization displayed reduced pigment contents, a response previously described as a protective mechanism to limit excess light absorption under P deficiency [79,80,81]. The higher carotenoid contents at 60 and 120 DAP observed in inoculated plants further suggest enhanced protection of the photosynthetic apparatus against photo-oxidative stress [82,83].

Among the treatments, AC showed the lowest ETR, φPSII, and *F_v_*/*F_m_* values, indicating greater susceptibility to photo-oxidative damage and reduced photochemical efficiency, underscoring the negative impact of P deficiency on photosynthetic performance. On the other hand, inoculated plants maintained higher ETR values at 60 and 180 DAP, reflecting the effective functioning of the electron acceptors in the biochemical phase of photosynthesis; the increase in ETR indicates a highly oxidized state of the quinone A (QA) acceptor, facilitating the use of excitation energy for electron transport. This process helps reduce the generation of reactive oxygen species, thereby preventing photo-oxidation [84,85,86].

Since ϕPSII is intrinsically linked to non-cyclic electron transport rates, the higher ϕPSII observed in *Bv* corresponds to increased ETR and enhanced photosynthetic rates. Along with *F_v_*/*F_m_*, ϕPSII is a reliable indicator of plant performance under various stress conditions [87,88]. This relationship explains why ETR emerged as a critical variable for shoot biomass production. Similar increases in ETR have been reported in crops inoculated with *Bacillus* spp., including pepper [89,90], sugar beet [91], sugarcane [92], and wheat [93].

The reduced *F_v_′*/*F_m_′* and qP values in AC suggest the accumulation of reduced QA in the PSII reaction center, impairing photochemical efficiency in leaves with lower P contents [81] and decreasing net CO_2_ assimilation. This finding corroborates earlier studies demonstrating the adverse effects of P deficiency on photosystems [80,81,94]. In contrast, the higher *F_v_′*/*F_m_′* and qP values observed in inoculated plants indicate greater directing of light energy to photochemistry, promoting ETR for carbon fixation, allowing most of the reducing power to be allocated to the carbon assimilation process, boosting biomass accumulation [95].

At 180 DAP, inoculated plants also showed 21.5% higher NPQ than non-inoculated plants. While NPQ is often interpreted as a sign of energy dissipation and reduced efficiency [96], our results demonstrate that elevated NPQ occurred in parallel with greater biomass, suggesting a protective role that sustained photochemical integrity under variable conditions. This is consistent with the notion that NPQ and qP jointly reduce O_2_ production in PSII antenna complexes, mitigating photo-inhibition [97,98,99].

The *F_v_*/*F_m_* ratio is a reliable indicator of photosynthetic performance, representing the maximum efficiency of light absorption by PSII for QA reduction [100,101]. Plants inoculated with *B. velezensis* had increased *F_v_*/*F_m_* values compared with non-inoculated plants, ranging from 0.80 to 0.86, values typical of healthy and non-stressed plants [95,102]. These results confirm that the UFV 3918 strain did not impair photosynthetic capacity but supported its maintenance. Similar increases in *F_v_*/*F_m_* after inoculation with PGPB have been reported in several crops [89,103,104].

Stomatal regulation balances CO_2_ uptake for photosynthesis and water loss through transpiration [105]. Stomatal conductance (*g_s_*) may be one of the main determinants of net CO_2_ assimilation (*A*) [106], and the rates of *g_s_* are determined by stomatal anatomical features, including density and size, and stomatal functional aspects [107]. Studies on *Arabidopsis* have shown that increased stomatal density (SD) can enhance gas exchange. Tanaka et al. [108] reported that higher SD increased *g_s_* and *A* under constant and saturated light conditions. Similarly, Sadoka et al. [109] observed that elevated SD accelerated *A* induction under fluctuating light, attributed to a higher initial *g_s_* value and a more rapid *g_s_* response during the early phase of photosynthetic induction.

*Bacillus velezensis* inoculation promoted an increase in SD, especially SDAB, resulting in greater *g_s_* and, consequently, higher *A*. Our findings align with those of Cappellari et al. [110], who reported increased SD in peppermint plants inoculated with *B. subtilis* GB03, and Silva et al. [111], who found that higher SDAB enhanced *A* in some sugarcane varieties under both hydrated and water-deficit conditions.

Intercellular CO_2_ concentration (*C_i_*) is critical for maximizing photosynthesis but depends on crop species and environmental conditions [112]. In our study, *C_i_* emerged as the most critical physiological variable for shoot biomass production, showing an inverse relationship with *A* and carboxylation efficiency (CE). The lowest *C_i_* values were observed in plants treated with *B. velezensis*, indicating that a higher proportion of CO_2_ was being assimilated. Similarly, Wang et al. [113] reported increased *C_i_* under lower P doses in cotton cultivars, with concurrent increases in *A* and reductions in *C_i_* under higher P availability.

Typically, higher *g_s_* values result in lower water use efficiency (WUE), potentially diminishing the benefits of increased photosynthetic performance for biomass production [108,114]. However, the balance between *A* and transpiration rate (*E*) was maintained despite the rise in *g_s_* induced by *B. velezensis*. This balance led to an increase in WUE at 60 DAP in inoculated plants. In contrast, no significant effect of the UFV 3918 strain on WUE was observed at 120 and 180 DAP, demonstrating the strain’s benefit to the photosynthetic apparatus.

The transient increase in WUE may be linked to *Bacillus*’s exopolysaccharide (EPS) secretion, which improves soil water retention [115,116,117]. Such mechanisms have also been associated with improved WUE in corn [118] and sugarcane propagated via pre-sprouted seedlings [119], correlating directly with higher root dry matter in inoculated plants. In sugarcane, *Bacillus* spp. have been shown to enhance CE by increasing *A* and the use of substomatal CO_2_ [38], highlighting the UFV 3918 strain’s role in improving CO_2_ flow to carboxylation sites and facilitating substrate metabolism for photoassimilate biosynthesis.

Most PSBs inhabit the rhizosphere, supported by root exudates derived from photosynthesis [120]. The enhanced CO_2_ assimilation and higher respiration (*R_d_*) in plants inoculated with *B. velezensis* is likely to have promoted microbial activity and energy supply, contributing to greater biomass accumulation through improved carbon balance [121,122,123,124].

The role of biochemical adjustments was equally evident. Although P is essential for protein synthesis [8], *B. velezensis* inoculation did not enhance protein content and even reduced it at high P doses. In contrast, its combination with low MAP doses increased total sugars, indicating that PSB helps maintain normal sugar metabolism under reduced P supply by ensuring adequate cytosolic phosphate (Pi) for sucrose synthesis [125]. Inoculated plants accumulated more total amino acids, compounds known to act as osmolytes that stabilize cellular metabolism under stress [126,127,128,129]. This aligns with reports that PSB inoculation enhances proline and related metabolites, supporting stress resilience.

Grasses respond to P deficiency with increased acid phosphatase activity in leaves, stems, and roots [130]. Inoculation with *B. velezensis* UFV 3918 increased leaf acid phosphatase activity by 16.4%, highlighting its phosphate-solubilizing potential and contribution to shoot biomass production. When combined with higher MAP doses, phosphatase activity decreased due to negative feedback from elevated cellular P levels, resulting from the high initial soil P content and phosphate fertilization [130,131]. It explains why this was the most important biochemical variable influencing shoot biomass production.

Phosphate-solubilizing bacteria release various organic acids capable of converting insoluble P forms into soluble ones. These organic acids chelate cations such as Al, Fe, and Ca, which are bound to P, using their hydroxyl and carboxyl groups to make P more accessible for plant uptake [132,133]. Additionally, these microorganisms facilitate the mineralization of organic P by producing hydrolytic enzymes, such as phosphatases, which catalyze the hydrolysis of phosphoester or phosphoanhydride bonds [44,134].

Enhanced P availability with *B. velezensis* inoculation was further supported by improved carboxylation efficiency, since net CO_2_ assimilation strongly depends on adequate P supply [8,135]. Biomass production behavior was strictly related to the soil P availability, which is confirmed by the high correlation of P in the soil with the variables stomatal density (0.80), photochemistry (0.88), gas exchange (0.83), photosynthetic pigments (0.89), leaf biochemistry (0.77) and, consequently, shoot biomass (0.92). Since more than 90% of crop biomass derives from photosynthetic products [136], these results underscore the central role of photosynthesis and respiration in sustaining growth. Consequently, the strong relationship between plant growth, photosynthesis, and respiration helps explain the higher shoot biomass verified in *Bv* and *Bv* + 1/3 MAP treatments.

Beyond P solubilization, PSBs promote plant growth through additional mechanisms, including the biosynthesis of phytohormones and secondary metabolites [137,138,139]. Specifically, *Bacillus* spp. have been reported to produce auxins, gibberellins, and expansins [140], which enhance plant growth and development. These mechanisms probably supported the enhanced shoot biomass observed with UFV 3918, particularly in combination with reduced MAP doses (*Bv* and *Bv* + 1/3 MAP). Several studies support the growth-promoting effects of PGPB, including PSBs, on sugarcane productivity; inoculation with PGPB enhanced both growth and yield [24,40,58,119,141].

Finally, it is important to note that this study was conducted in a greenhouse using pots. Such controlled conditions allow a detailed understanding of plant–microbe interaction mechanisms. To confirm the broader applicability and agronomic relevance of UFV 3918, future studies under field conditions, which present greater variability in soil, climate, and microbial populations, are warranted.

In summary, inoculation with *B. velezensis* UFV 3918, either alone or with reduced MAP doses, enhanced phosphate solubilization efficiency, improved photosynthetic performance, and regulated cellular metabolism, culminating in increased sugarcane biomass. These findings demonstrate the potential of this strain to optimize sugarcane production under lower P inputs, while reinforcing the need for field validation.

## 4. Materials and Methods

### 4.1. Cultivation Conditions, Plant Material, Experimental Design, and Treatments

The experiment was carried out between November 2021 and May 2022 in a greenhouse at the Department of Crop Production, School of Agricultural Sciences—FCA/UNESP, located in Botucatu, São Paulo, Brazil (22°51′01” S, 48°25′55” W, 786 m above sea level).

**Figure 12:**
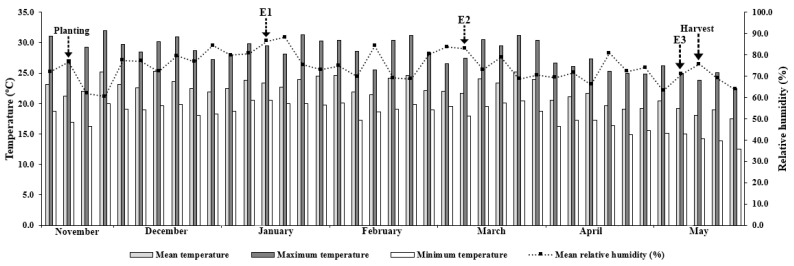
Average, maximum, and minimum air temperature and relative humidity inside the protected environment during the experimental period. Adapted from Santos et al. [58]. E1: 1st evaluation (60 DAP); E2: 2nd evaluation (120 DAP); E3: 3rd evaluation (180 DAP).

Temperature and humidity data were continuously monitored using a data logger (Instrutherm, HT-500, São Paulo, SP, Brazil). During the experiment, the air temperature inside the greenhouse ranged from 12.5 to 32.0 °C, with an average of 21.2 °C, 23.4 °C, 21.6 °C, 19.2 °C, and 18.0 °C at planting, 1st evaluation (E1), 2nd evaluation (E2), 3rd evaluation (E3), and harvest, respectively (Figure 12). Relative humidity during the cultivation cycle ranged from 60.4 to 88.1%, with an average of 76.5%, 86.4%, 82.9%, 70.9%, and 75.6% at planting, E1, E2, E3, and harvest, respectively (Figure 12).

Plants were irrigated using a drip system (Netafim, PCJ-CNL 4 L/h, Ribeirão Preto, SP, Brazil), maintaining soil moisture at 90% of the pot’s water retention capacity. The water regime was monitored using a portable moisture meter (5TM ProCheck, Decagon Devices, Inc., Pullman, WA, USA).

According to granulometric analysis, the soil used was a dystrophic red latosol [142], characterized by a medium texture with 68.2% sand, 25.7% clay, and 6.1% silt. Solarization was employed to eliminate pathogens [143], minimizing interference from other microorganisms in plant development and phosphate solubilization.

After solarization and before treatment application, the soil had a pH (CaCl_2_) of 6.0, organic matter content of 40.1 g dm^−3^, and low exchangeable acidity (Al^3+^ = 0.7 mmol_c_ dm^–3^), with potential acidity (H + Al) totaling 20.8 mmol_c_ dm^–3^. The exchangeable potassium (K), calcium (Ca), and magnesium (Mg) concentrations were 0.6, 75.2, and 26.4 mmol_c_ dm^−3^, respectively. The sum of bases (SB) was 102.2 mmol_c_ dm^−3^, and the cation exchange capacity (CEC) reached 123.0 mmol_c_ dm^−3^, resulting in a base saturation (V%) of 83.0%. Available phosphorus (P_resin_) was 61.8 mg dm^−3^, and sulfur (S) was 34.0 mg dm^−3^. Micronutrient concentrations were: copper (Cu) = 0.3, iron (Fe) = 22.7, manganese (Mn) = 0.7, zinc (Zn) = 1.2, and boron (B) = 0.2 mg dm^−3^.

Soil fertility was adjusted based on chemical analysis [144], with fertilizers incorporated into the soil at planting. Different fertilization strategies were adopted using varying doses of monoammonium phosphate (MAP, containing 60% soluble P in neutral ammonium citrate and 12% N), combined with fixed rates of KCl and urea. The recommended MAP dose treatment received 125 kg ha^−1^ of MAP (2.8 g pot^−1^), 250 kg ha^−1^ of KCl (5.635 g pot^−1^), and 36.12 kg ha^−1^ of urea (0.813 g pot^−1^). The 2/3 MAP treatment received 83.33 kg ha^−1^ of MAP (1.878 g pot^−1^), 46.3 kg ha^−1^ of urea (1.04 g pot^−1^), and the same KCl dose. The 1/3 MAP treatment received 41.7 kg ha^−1^ of MAP (0.939 g pot^−1^), 56.47 kg ha^−1^ of urea (1.272 g pot^−1^), and 250 kg ha^−1^ of KCl. The treatment without MAP received only urea (66.6 kg ha^−1^; 1.5 g pot^−1^) and KCl (250 kg ha^−1^; 5.635 g pot^−1^), without phosphorus addition at planting. Additionally, urea was top-dressed at 1.5 g pot^−1^ (equivalent to 30 kg N ha^−1^) before stalk formation, as recommended for medium-textured soils [144].

The sugarcane variety RB966928 was chosen for its extensive cultivation in Brazil, accounting for 17.7% of the planted area in São Paulo [145]. This variety is recognized for its rapid growth, robust sprouting, high tillering capacity, superior yields, and overall plant health.

A completely randomized design was used, consisting of six treatments: absolute control (AC, without MAP); commercial control (CC, recommended MAP dose or 3/3 MAP); *Bacillus velezensis* UFV 3918 (*Bv*); *Bv* + 1/3 MAP; *Bv* + 2/3 MAP; *Bv* + 3/3 MAP, with four replicates. Healthy buds of uniform size (approximately 5 cm long and 2 cm in diameter) were selected for sprouting standardization. Five buds were planted per 50 L pot containing 45 dm^3^ of soil. After sprouting, plants were thinned to one per pot to ensure uniformity.

Planting was carried out on 12 November 2021, with fertilizer applied at sowing. Bacterial inoculation consisted of preparing a solution containing 75 mL of water (pH 7.0) and 2 mL of a commercial formulation of *B. velezensis* strain UFV 3918 (1.0 × 10^8^ CFU mL^−1^; 7 g L^−1^). Each bud received 15.4 mL of this solution in bacterial treatments, while non-bacterial treatments included the same volume of water. The commercial formulation, recommended at a field application rate of 2 L ha^−1^ for sugarcane cultivation, was provided by Vittia (São Joaquim da Barra, São Paulo, Brazil).

### 4.2. Physiological Assessments

Physiological variables, including stomatal density, gas exchange, and chlorophyll *a* fluorescence, were assessed at 60, 120, and 180 days after planting (DAP) using the +1 leaf. This leaf, also referred to as the TVD leaf (top visible dewlap), is the first fully expanded leaf with a visible ligule and is considered the most photosynthetically active [146].

Stomatal counts were conducted using epidermal impressions obtained by applying a thin layer of clear nail polish to the abaxial and adaxial surfaces of the +1 leaf, parallel to the midrib. After drying, the nail polish was lifted with transparent adhesive tape, which was mounted on slides for analysis under an optical microscope (Biovideo, BEL Photonics, Monza, Italy) at 40× magnification. Stomatal counts were performed in an area of 0.0744 mm^2^ following the protocol of Mazumdar et al. [147].

Gas exchange variables—net CO_2_ assimilation rate (*A*), stomatal conductance (*g_s_*), transpiration rate (*E*), and intercellular CO_2_ concentration (*C_i_*)—were measured in the central portion of the +1 leaf using an infrared gas analyzer (IRGA) (LI-COR Biosciences Inc., LI-6400XT, Lincoln, NE, USA). The leaf chamber was equipped with an artificial LED light source (6400-40 LCF, LI-COR; 90% red and 10% blue spectra) providing a photosynthetic photon flux density (PPFD) of 1500 μmol photons m^–2^ s^–1^, based on a previously established light-response curve. Measurements were performed between 9:00 am and 11:30 am under ambient CO_2_ concentration, temperature, and humidity conditions. Instantaneous water use efficiency (WUE) was calculated as the *A*/*E* ratio, and instantaneous carboxylation efficiency (CE) was calculated as the *A*/*C_i_* ratio.

Chlorophyll *a* fluorescence and respiration (*R_d_*) were measured at night (7:30 pm to 11:00 pm). Respiration was evaluated at sufficiently low irradiance (<1 μmol m^−2^ s^−1^) under ambient CO_2_, temperature, and humidity. Fluorescence variables were assessed using a 6400-40 leaf chamber fluorometer coupled to the IRGA. For light-adapted leaves, maximum fluorescence (*F_m_′*) was determined with a saturation pulse of 7000 μmol photons m^−2^ s^−1^ for 0.8 s, while actinic light was set at 200 μmol photons m^−2^ s^−1^. Dark-adapted maximum fluorescence (*F_m_*) was obtained after a saturation pulse of 4200 μmol photons m^−2^ s^−1^ for 0.8 s, while minimum fluorescence (*F_0_*) was measured under <1 μmol m^−2^ s^−1^ irradiance. Potential quantum yield of PSII (*F_v_′*/*F_m_′*), maximum variable quantum yield of PSII (*F_v_*/*F_m_*), effective quantum yield of linear electron flow through PSII (φPSII), photochemical quenching (qP), non-photochemical quenching (NPQ), and relative electron transport rate (ETR) were calculated according to Schreiber et al. [148].

### 4.3. Biochemical Assessments

The contents of photosynthetic pigments (chlorophylls *a*, *b*, total, and carotenoids) were measured at 60, 120, and 180 days after planting (DAP). Two leaf discs (0.28 cm^2^ each) were collected from the +1 leaf using a punch, avoiding the midrib and leaf edges. The discs were immersed in dimethylformamide (DMF) for 48 h and protected from light. Subsequently, 1 mL of the pigment extract was diluted in 1 mL of deionized water, and absorbance readings were taken using a spectrophotometer (Shimadzu, UV-2700, Kyoto, Japan) at wavelengths of 480, 647, and 664 nm. Pigment concentrations were calculated using the method by Wellburn [149], and results were expressed in μg cm^−2^.

For the analysis of protein content, soluble sugars, amino acids, and acid phosphatase activity, +1 leaves were collected one week before harvest, frozen in liquid nitrogen, and stored at −80 °C (NUAIRE Inc., NU-9668GC, Plymouth, MN, USA). Soluble protein content was determined from 100 mg of leaf tissue macerated in liquid nitrogen and homogenized in 0.1 M potassium phosphate buffer (pH 6.8) containing 0.1 mM ethylenediamine tetraacetic acid, 1 mM phenylmethylsulfonyl fluoride, and 200 mg of polyvinylpyrrolidone. The homogenate was centrifuged at 5000× *g* for 10 min at 4 °C, and soluble proteins were quantified by mixing 20 μL of the supernatant with 5 mL of Coomassie Brilliant Blue G-250 solution. After 15 min, absorbance was measured at 595 nm (Shimadzu UV-2700, Kyoto, Japan), and protein concentrations were determined using a bovine serum albumin standard curve (1 mg mL^−1^). Results were expressed as mg g^−1^ fresh matter (FM) [150].

Soluble sugars were determined using 20 mg of freeze-dried leaf tissue. Samples were extracted in 4 mL of deionized water with stirring for 1 h, followed by centrifugation at 3000× *g* for 15 min. The supernatant was re-centrifuged at 6000× *g* for 10 min. A 0.5 mL aliquot of extract was mixed with 0.5 mL of 5% phenol (*v*/*v*) and 2.5 mL of sulfuric acid, stirred, and cooled in an ice tray. Absorbance readings were taken at 490 nm (Shimadzu, UV-2700), and concentrations were calculated following Dubois et al. [151]. Results were expressed in mg g^−1^ FM^−1^.

Total amino acids were determined using 20 mg of freeze-dried leaf tissue homogenized in 2 mL of 0.01 M Na-K-phosphate buffer (pH 7.6) with 0.1 M NaCl. After stirring for 1 h in trays with ice and centrifugation at 3000× *g* at 4 °C for 5 min, 1 mL of the supernatant was mixed with 1 mL of 10% (*w*/*v*) trichloroacetic acid and left for 1 h. The mixture was centrifuged at 12,000× *g* at 4 °C for 5 min, and the supernatant was used as a crude extract. A 0.5 mL aliquot was mixed with 0.25 mL of 0.2 M sodium citrate buffer (pH 5.0), 0.1 mL of 5% ninhydrin in 100% methylcellosolve (*v*/*v*), and 0.5 mL of 0.0002 M potassium cyanide in methylcellosolve (*v*/*v*). Samples were heated in a water bath at 100 °C for 20 min, cooled, and diluted with 3.65 mL of 60% ethanol (*v*/*v*). Absorbance was measured at 570 nm (Shimadzu, UV-2700) and compared with a glycine standard curve (0.1–1.0 μmol mL^−1^) [152]. Results were expressed in μmol g^−1^ FM^−1^.

Acid phosphatase activity (AP-EC 3.1.3.2) was measured in 500 mg of leaf tissue macerated in liquid nitrogen and homogenized in 0.1 M sodium acetate buffer (pH 5.6). Homogenates were centrifuged at 15,000× *g* at 4 °C for 20 min, and crude extracts were used. Enzyme activity was initiated by incubating 500 μL of extract with 200 μL of substrate (2 mM disodium *p*-nitrophenyl phosphate in 150 mM sodium acetate buffer, pH 5.6) at 37 °C for 10 min. The reaction was stopped with 300 μL of saturated Na_2_CO_3_. Absorbance was read at 400 nm (Shimadzu, UV-2700), and activity was expressed in nmol *p*-nitrophenyl phosphate (pNPP) min^−1^ mg^−1^ protein [153].

### 4.4. Phosphorus Content in the Soil and Shoot

Soil samples were collected at 0–0.15 m depth in May 2022. The sampled soil was then dried in a forced-air oven at 40 °C for 96 h and passed through a 2 mm sieve. Phosphorus (P) concentrations in the soil were analyzed according to van Raij et al. [154] methodology, extracted using ion exchange resin, and determined by spectrophotometry.

Nutrient diagnosis consisted of determining the nutrient content in samples of diagnostic leaves, i.e., +1 leaves [155,156]. In sugarcane, the +1 leaf is characterized as the first leaf with a fully open ligule, also known as the TVD leaf (top visible dewlap) [146].

At 180 DAP, the median portions of the +1 leaves were collected, discarding the central vein, the leaf sheaths of the +1 leaves, and the median portions of the main stalks. The sampled material was placed in a forced-air circulation oven at 60 °C until it reached a constant weight and then ground in a Willey mill. P was extracted by nitroperchloric digestion [157] and determined by spectrophotometry.

Shoot P accumulation was calculated using Formula (1), as follows:

(1) PAc=SB×PC

where PAc is phosphorus accumulation (g plant^−1^), SB is shoot biomass (g), and PC is phosphorus concentration (g kg^−1^).

### 4.5. Shoot Biomass

At 180 days after planting (DAP), the plants were harvested and partitioned into leaves, leaf sheaths, stalks, and roots. Shoot biomass (SB) was calculated as the sum of the biomass of leaves, leaf sheaths, and stalks. Samples were dried in a forced-air circulation oven at 65 °C until reaching constant mass and subsequently weighed using a precision scale with 0.01 g accuracy (Balmak, ELC-6/15/30, Santa Bárbara d’Oeste, SP, Brazil).

### 4.6. Statistical Analysis

Data were tested for normality using the Shapiro–Wilk test and homoscedasticity using the Levene test. Once these assumptions were satisfied, analysis of variance (ANOVA) was performed using the F test, followed by Tukey’s test (*p* ≤ 0.05) for mean comparison. Statistical analyses were conducted using AgroEstat software (AgroEstat, version 2015, Jaboticabal, SP, Brazil). Biochemical and yield variables were further analyzed through regression adjustments to assess the effects of MAP doses associated with *Bacillus velezensis* (*Bv*, *Bv* + 1/3 MAP, *Bv* + 2/3 MAP, and *Bv* + 3/3 MAP) using Minitab software (Minitab^®^, version 19, State College, PA, USA).

Variables were organized into five distinct groups: stomatal density (SDAD and SDAB); photochemistry (ETR, *F_v_′*/*F_m_′*, φPSII, qP, NQP, and *F_v_*/*F_m_*); gas exchange (*A*, *g_s_*, *C_i_*, *E*, Rd, WUE, and CE); photosynthetic pigments (Chl*_a_*, Chl*_b_*, total Chl, Chl*_a_*/Chl*_b_*, and carotenoids); leaf biochemistry (protein, total sugars, total amino acids, and leaf acid phosphatase); and biomass (SB). Principal component analysis (PCA) was applied to each variable group using the nonlinear iterative partial least squares (NIPALS) algorithm [158]. The minimum number of components explaining at least 70% of the total variability was selected for each group. The PCA scores from the five variable groups were compared with shoot biomass scores and represented in scatter plots, averaging values for each treatment. Pearson’s linear correlation coefficient (*p* < 0.01) was used to evaluate the relationship between each variable group’s main components, phosphorus in the soil and the shoot, and the sugarcane plants’ shoot biomass.

## 5. Conclusions

The inoculation of sugarcane buds with *B. velezensis* UFV 3918, either alone or combined with reduced doses of MAP (*Bv*, *Bv* + 1/3 MAP, and *Bv* + 2/3 MAP), led to an increase in P availability, which strongly correlated with physiological and biochemical variables. These treatments enhanced stomatal density, photosynthetic pigment contents, and relative electron transport rate. These factors improved net CO_2_ assimilation and carboxylation efficiency, increasing shoot biomass production. Additionally, the treatments boosted sugar and total amino acid contents and leaf acid phosphatase activity, reflecting the regulation of cell metabolism and the potential for P solubilization. In this study, the high initial soil P content allowed inoculation with *B. velezensis* to reduce phosphate fertilization without compromising the crop’s photosynthetic or productive performance. However, there was a tendency for production to decline when the strain was combined with higher MAP doses, suggesting that *Bv* + 1/3 MAP is the most suitable combination. This research demonstrates that inoculating sugarcane with *B. velezensis* UFV 3918 is a viable strategy for optimizing P use in sugarcane, supporting future research to reduce the use of phosphate fertilizers.

## Figures and Tables

**Table 1:** Phosphorus (P) content in the soil and shoot P accumulation in sugarcane, under treatments with and without inoculation of *Bacillus velezensis* UFV 3918 (*Bv*) and doses of mono ammonium phosphate (MAP), after 180 days of cultivation.

Treatments	P in the Soil (mg dm^–3^)	P Accumulated in Sugarcane (g plant^−1^)
AC	24.95 d	0.62 c
CC	33.30 c	0.72 b
*Bv*	42.02 a	0.80 a
*Bv* + 1/3 MAP	39.22 ab	0.72 b
*Bv* + 2/3 MAP	37.00 bc	0.75 ab
*Bv* + 3/3 MAP	41.01 a	0.75 ab
C.V. (%)	6.61	5.10
Regression	Y = 2.5 × 10^–5×3^ − 2.21 × 10^–3×2^ − 0.0389x + 42.02 (R^2^ = 0.70)	Y = −1 × 10^–6^x^3^ + 1.18 × 10^–4^x^2^ − 5.52 × 10^–3^x + 0.798 (R^2^ = 0.70)

Averages followed by the same letter do not differ according to Tukey’s test at 5% probability. The regression equations and R^2^ refer to the association between *Bv* and MAP doses at a 5% significance level. AC: absolute control (without MAP); CC: commercial control (3/3 MAP—100% of recommended MAP dose, without *Bv*); C.V.: coefficient of variation.

**Table 2:** Explanation of the percentage of the first component of variables: stomatal density, photochemistry, gas exchange, photosynthetic pigments, and leaf biochemistry.

Groups of Variables	Explanation Percentage (%)
Stomatal density	72.98
Photochemistry	84.64
Gas exchange	79.07
Photosynthetic pigments	82.32
Leaf biochemistry	98.29

## Data Availability

Once all the data have been published, the data supporting this study’s findings will be available in a repository [UNESP] at http://hdl.handle.net/11449/242558 (accessed on 19 August 2025), reference number S237s. The data are available from the corresponding author upon reasonable request.
